# Self-completion method of endoscopic submucosal dissection using the Endosaber for treating colorectal neoplasms (with video)

**DOI:** 10.1038/s41598-022-09792-8

**Published:** 2022-04-06

**Authors:** Mitsuru Esaki, Shun Yamakawa, Ryoji Ichijima, Sho Suzuki, Chika Kusano, Hisatomo Ikehara, Yosuke Minoda, Eikichi Ihara, Takuji Gotoda

**Affiliations:** 1 Division of Gastroenterology and Hepatology, Department of Medicine, Nihon University School of Medicine, 1-6 Kanda-Surugadai, Chiyoda-ku, Tokyo, 101-0062 Japan; 2 Department of Medicine and Bioregulatory Science, Graduate School of Medical Sciences, Kyushu University, 3-1-1 Maidashi, Higashi-ku, Fukuoka, 812-8582 Japan; 3 Department of Gastroenterology, Kitasato University School of Medicine, 1-5-1 Kitasato, Minami-ku, Sagamihara, 252-0375 Japan; 4 Department of Gastroenterology and Metabolism, Graduate School of Medical Sciences, Kyushu University, 3-1-1 Maidashi, Higashi-ku, Fukuoka, 812-8582 Japan

**Keywords:** Gastrointestinal cancer, Colorectal cancer, Cancer, Gastroenterology

## Abstract

Endoscopic submucosal dissection (ESD) is effective for the treatment of colorectal neoplasms. We have developed a self-completion ESD (S-ESD) using Endosaber without requiring additional instruments or assistance. This prospective cohort study was conducted to investigate the feasibility of S-ESD for colorectal neoplasms. Patients with colorectal neoplasms measuring 20–40 mm in size were enrolled. A single operator, without assistance, performed ESD using only the Endosaber. The primary outcome was the success rate of S-ESD. Secondary outcomes included procedure time, the rates of en bloc, complete, and curative resection, and complication rates, including the incidence of perforation and delayed bleeding. In total, 15 patients with 15 lesions were enrolled. The median size of the resected lesions was 28 mm (interquartile range 25–29 mm). S-ESD success rate of 100% was achieved. The median procedure time was 44 min (29.5–53.5 min). We observed en bloc, complete, and curative resection rates of 100%, 93.3%, and 86.7%, respectively, and a complication rate of 6.7% (perforation: 0%, delayed bleeding: 6.7%). S-ESD for colorectal neoplasms was successfully performed with favorable treatment outcomes and low complication rates. S-ESD reduces the number of devices and extent of assistance, making S-ESD a simple and cost-effective procedure.

## Introduction

Endoscopic resection has been developed as a treatment for superficial gastrointestinal neoplasms, including colorectal neoplasms^1^. Initially, endoscopic mucosal resection (EMR) was used for treating colorectal lesions. Later, endoscopic submucosal dissection (ESD) was developed as a local treatment for gastric neoplasms and, subsequently, has been used for treating colorectal neoplasms^2^. ESD allows complete resection and accurate histological diagnosis for early colorectal neoplasms, thereby contributing to the prevention of tumor recurrence^1,3,4^. According to Japanese guidelines, ESD is recommended for colorectal lesions that are ≥ 20 mm because such lesions are difficult to manage via en bloc resection with EMR^5,6^.

The conventional ESD procedure includes submucosal injection, mucosal incision, submucosal dissection, and hemostasis. Injection needle, endo-knife, and hemostatic forceps are used during these procedures and are manipulated by the assistant^2^. The assistant’s skill level may affect treatment outcomes. Therefore, successful ESD requires not only a well-trained operator but also a well-trained assistant. However, it is not always possible to ensure this combination of well-trained personnel. In addition, conventional ESD is more expensive than EMR^7,8^. ESD is currently required to be performed such that the cost is minimized as much as possible while ensuring adequate treatment outcomes.

**Figure 1:**
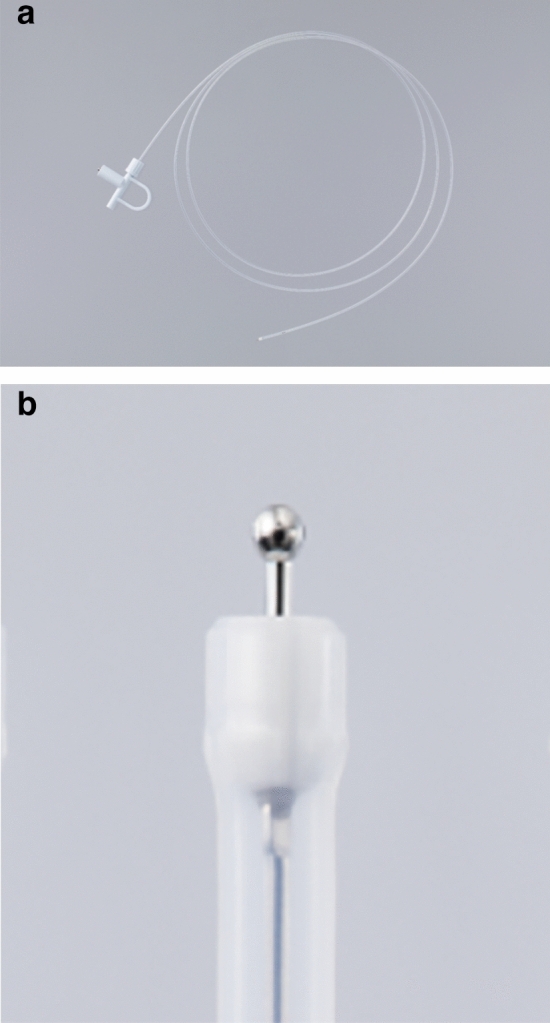
Photographs of the Endosaber (Sumitomo Bakelite, Tokyo, Japan). (**a**) A photo of the entire Endosaber. (**b**) A close-up photograph of the ball-tip on the distal end of the Endosaber.

Recently, we invented a technique known as self-completion ESD (S-ESD) using the Endosaber (Sumitomo Bakelite, Tokyo, Japan; Fig. 1) as a more advanced ESD procedure^9^. The Endosaber can perform all ESD procedures, including injection, cutting, and coagulation. Thus, S-ESD can be performed by a single endoscopist using only the Endosaber, thereby eliminating the need for other endoscopic devices and staff assistance, thus reducing both the burden on the medical staff and the cost compared with conventional ESD. We have previously demonstrated the feasibility of S-ESD on mock lesions in ex vivo porcine models^10^. However, S-ESD has never been considered in human clinical studies. Therefore, we conducted a prospective study to investigate the feasibility of S-ESD for the treatment of patients with colorectal neoplasms.

## Results

### Characteristics of enrolled patients

**Figure 2:**
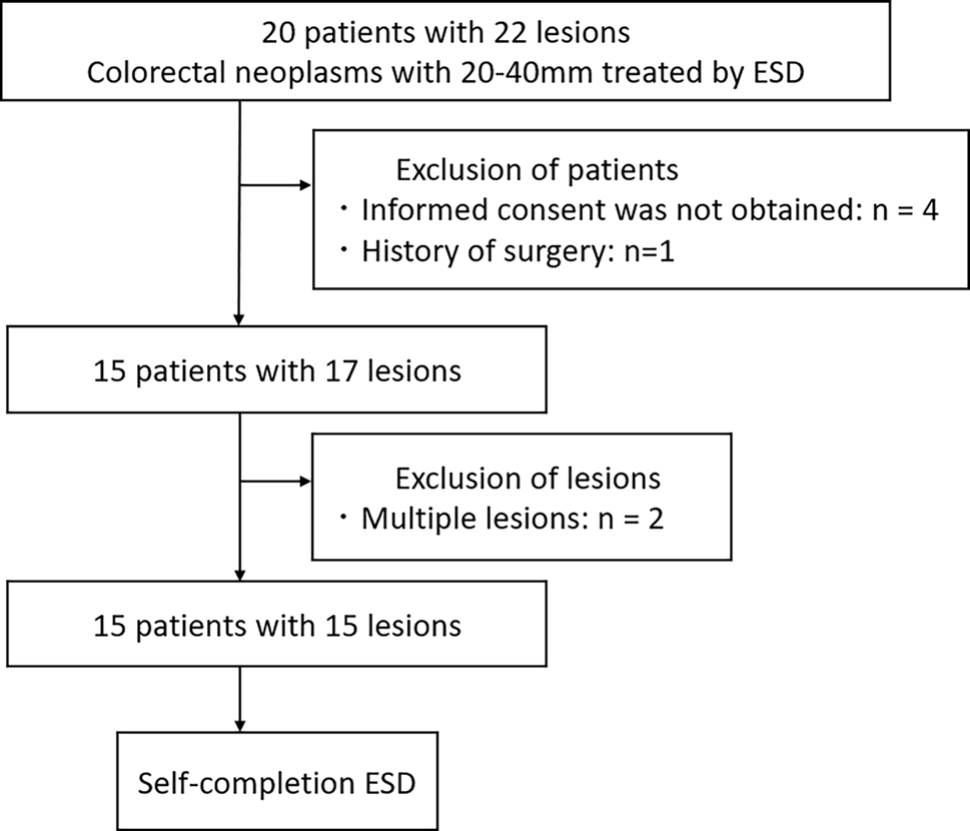
The flowchart of enrolled patients in this study. ESD, endoscopic submucosal dissection.

A flowchart of the enrolled patients is shown in Fig. 2. During the study period, 20 patients with 20–40 mm colorectal lesions met the inclusion criteria. Informed consent for this study could not be obtained in four cases. One patient had a history of rectal amputation, and such patients were excluded from this study. In addition, two patients had double lesions indicated for ESD, wherein the larger lesion was treated by S-ESD, and the other was treated by conventional ESD. Finally, 15 patients with 15 lesions were included in this study. The patients’ background characteristics are shown in Table 1, and the median tumor size was 28 [25–29] mm. Only one lesion (6.7%) was classified as submucosal invasion in terms of the estimated tumor depth.

**Table 1:** Characteristics of enrolled patients and lesions.

		N = 15
Age	Median [IQR]	77 [63–81]
Sex	Male/female	10/5
Tumor location	Ce/A/T/D/S/R	0/7/3/1/2/2
Tumor size	Median, mm [IQR]	28 [25–29]
Estimated tumor depth	Mucosa/submucosa	14/1

IQR, interquartile range.

Ce, cecum; A, ascending colon; T, transverse colon; D, descending colon; S, sigmoid colon; R, rectum.

### Treatment outcomes

The treatment outcomes are summarized in Table 2. All the S-ESDs were completed with the Endosaber, without the use of any other devices or need for assistants. Therefore, the success rate of the S-ESD was 100% (15/15). The en bloc resection, complete resection, and curative resection rates were 100% (15/15), 93.3% (14/15), and 86.7% (13/15), respectively. The one incompletely resected lesion was an adenoma in the ascending colon that was 26 mm in size. While the S-ESD was completed and en bloc resection was achieved for this lesion, the histological examination showed that the horizontal margin was positive for colorectal neoplasm. Similarly, there was another case of non-curative resection. In this case, the lesion was an adenocarcinoma in the rectum that was 30 mm in size. As per our methodology, the lesion was treated with complete resection; however, the histological assessment showed a differentiated adenocarcinoma with a submucosal invasion depth of 2000 µm from the muscularis propia. No lymphovascular infiltration was observed, and it had a budding grade of 1. No additional treatment was provided in this case according to the patient’s wish, and no recurrence occurred within 1 year of the endoscopic treatment.

**Table 2:** Treatment outcomes of S-ESD for colorectal neoplasms.

		N = 15
Self-completion	n (%)	15/15 (100)
Procedure time	Median [IQR]	44 [29.5–53.5]
En bloc resection	n (%)	15/15 (100%)
Complete resection	n (%)	14/15 (93.3%)
Curative resection	n (%)	13/15 (86.7%)
Complication	n (%)	1/15 (6.7%)
Perforation	n (%)	0/15 (0%)
Delayed bleeding	n (%)	1/15 (6.7%)
Resected specimen size	Median, mm [IQR]	35 [31.5–38]
Histology	Cancer/adenoma	9/6
Tumor depth	Mucosa/submucosa	14/1

S-ESD, self-completion endoscopic submucosal dissection.

**Figure 3:**
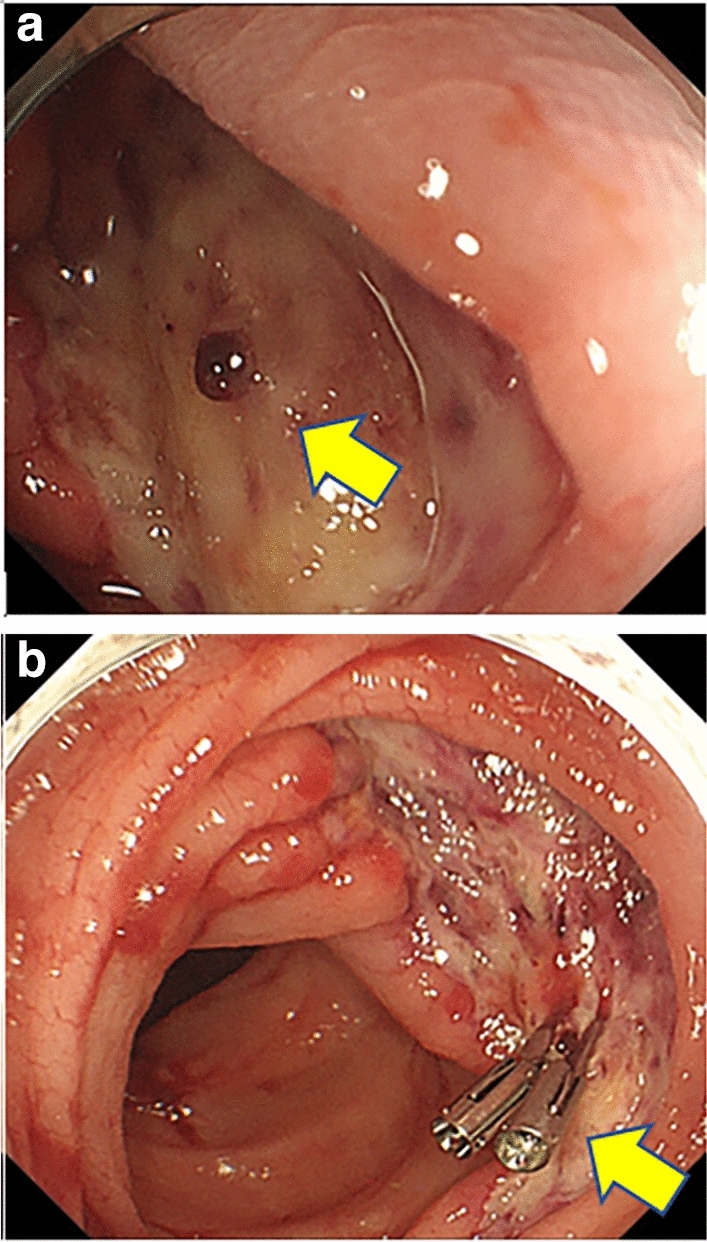
The images of a follow-up colonoscopy due to delayed bleeding. (**a**) Visible vessel on the artificial mucosal defect. (**b**) Hemoclips placed on the vessel.

Further, we observed a complication rate of 6.7% (1/15). Delayed bleeding occurred in one case despite an absence of perforation. The patient was an 81-year-old woman with a 35-mm laterally spreading tumor in the transverse colon. En bloc resection was achieved, and no complications occurred during the procedure. However, hematochezia was observed at one and two days post-ESD. In addition, a 4.1 g/dl decrease in the hemoglobin level (from 12.9 to 8.8 g/dl) was detected in a blood test. In response, endoscopy was performed under bowel preparation on the second day after the S-ESD. The endoscopy revealed no active bleeding; however, a vessel located on an artificial ulcer made with the S-ESD was observed (Fig. 3a). Hemoclips were placed on the visible vessel to prevent re-bleeding (Fig. 3b). Subsequently, no re-bleeding occurred, and the patient was later discharged.

## Discussion

In this study, we have shown for the first time that S-ESD using the Endosaber could be applied for the local treatment of colorectal neoplasms between 20 and 40 mm in size. All procedures of colorectal S-ESDs were performed successfully without the need of any other ESD devices or help from assistants in a cohort study comprising patients with 20–40-mm colorectal neoplasms, achieving favorable treatment and histological outcomes with low complication rates.

In conventional ESD, each procedure requires the cooperation of the operator and the assistant manipulating the endoscope and the endoscopic devices, respectively. Therefore, the treatment outcomes of conventional ESD may depend not only on the operator’s skill but also on the assistant’s skill. Furthermore, the operator is required to instruct the assistant at each step of the procedure. Even if each instruction is short, their accumulation may lead to loss of time and extension of the total procedure time. Communication errors may equally lead to complications during procedures. If the assistant is insufficiently skilled, they may not fully understand the operator’s instructions. On the contrary, in S-ESD using Endosaber, the operator can perform each procedure without distraction and delay. There is no need to consider communication errors between the operator and the assistant. In addition, S-ESD allows the assistant to view the total ESD procedure objectively by eliminating the need for assistance in using the endoscopic devices. This allows the assistant to focus on monitoring the patient’s condition, including vitals, which can contribute to the prevention of ESD-related complications. In this study, all procedures of S-ESD could be completed using Endosaber, without the use of any other devices or the need for assistants, indicating that S-ESD is feasible for treatment of colorectal neoplasms.

The colorectal S-ESD presented in this study could not be achieved without the development of the Endosaber. The Endosaber enables the performance of several steps and procedures by the operator as follows: First, the solution can be injected into the submucosal layer using the water jet system, which is connected to the Endosaber^10,11^. Second, pre-cutting the mucosa before the first injection eliminates the need for the injection usually performed by the medical staff in the ESD procedure. Third, hemostasis for active bleeding and prophylactic coagulation can be performed via the ball-tip of the Endosaber without requiring conventional hemostatic forceps. Furthermore, the attachment of a ball-tip to the endo-knife has been reported to improve its operability and increase hemostatic efficacy during the ESD procedure^12^. Finally, the Endosaber is a fixed endo-knife with a ball-tip at the distal end, eliminating the need to insert, withdraw, open, or close it; this is a unique characteristic of the Endosaber. In addition to the characteristics of the Endosaber, one benefit of ESD using the Endosaber is that it can contribute to decreased medical costs. Not only does the Endosaber cost less than other endo-knives but it also perform multiple roles in the ESD procedure. ESD using the Endosaber may be more cost-effective than conventional ESD.

Regarding the efficacy of colorectal S-ESD using the Endosaber, satisfactory treatment and histological outcomes were achieved in this study. A previous meta-analysis showed that the conventional colorectal ESD has a 92% en bloc resection rate, an 83% complete resection rate, and an 86% curative resection rate^13^. In this study, the en bloc resection rate, complete resection rate, and curative resection rate were 100% (15/15), 93.3% (14/15), and 86.7% (13/15), respectively. The two less successful cases included one lesion with incomplete resection and another with a submucosal invasion depth of > 1000 µm. Despite possible differences in the histological assessment and background characteristics of the patients between this study and previous studies of conventional ESD, colorectal S-ESD using the Endosaber achieved satisfactory treatment and histological outcomes comparable to the conventional colorectal ESD.

Concerning complications associated with colorectal S-ESD using the Endosaber, we observed low complication rates in this study. Intraoperative and delayed perforation incidence rates of 4.2% and 0.22%, respectively, have been reported in conventional colorectal ESD procedures, in contrast to the absence of procedure-related perforations observed with S-ESD in this study. The dull tip of the Endosaber allowed us to perform the initial mucosal pre-cut without administering a submucosal injection although the pre-cut was made with the Endosaber tilted as much as possible to avoid affecting the muscle layer. Once a small portion of the submucosal layer was exposed by the initial mucosal pre-cut, a sufficient volume of viscous Glyceol solution could be injected into the submucosal layer using the water jet system of the Endosaber. The thickened submucosal layer could be confirmed by the blue color of the injected solution with a small amount of indigo carmine dye. Subsequently, submucosal dissection was performed by hooking the ball-tip of the endo-knife to the submucosal tissue and sweeping it off into the lumen, which allowed for coagulation and the incision to be at a safe distance from the muscle layer. As such, our technique can prevent injury and thermal damage to the muscle layer, which may be the reason for the lack of intraoperative or delayed perforation.

In contrast, bleeding is another problematic complication of ESD^14^. The immediate and delayed major bleeding incidence rates are reportedly 0.75% and 2.1%, respectively. Although uncontrollable major bleeding did not occur in S-ESD using the Endosaber in this study, one case was accompanied by delayed bleeding. Hemostasis for active bleeding and prophylactic coagulation for visible vessels during the S-ESD could be conducted with the Endosaber. The ball-tip at the distal end of the endo-knives included in the Endosaber assist in coagulation^12^. The patient with delayed bleeding was taking antithrombotic drugs, which are known to be significant risk factors for delayed bleeding^15,16^. Although S-ESD can be performed in patients taking antithrombotic drugs according to the recommendations and guidelines for gastroenterological endoscopy in patients undergoing antithrombotic treatment, colonoscopy and hemostasis was required due to repeated bloody stools after S-ESD^17^. In summary, the risk of complications associated with S-ESD using the Endosaber appears to be equivalent to that of conventional ESD.

Although all procedure of colorectal S-ESDs can be performed successfully without any significant complications or assistance, it is important that the S-ESD be accompanied by a medical assistant to ensure that other devices, including hemostatic forceps and hemoclips, are accessible in case of major complications, including a massive bleeding, that cannot be controlled by the Endosaber and intraoperative perforation. This is a limitation of the current S-ESD using the Endosaber. Several endoscopic procedures have been previously performed by at least two operators but are currently performed by one operator in some situations: colonoscopy, double-balloon endoscopy, and cannulation of endoscopic retrograde cholangiopancreatography^18,19^. In colonoscopy, especially, the self-insertion method has been standard due to the advances in endoscopic instrumentation. These concepts can be applied to ESD. This was a pilot study of S-ESD using the Endosaber. S-ESD can be performed by only one operator in the future if new devices capable of managing complications by self-manipulation are developed, including hemostatic forceps for bleeding and clips for intraoperative perforation.

This study has several limitations. First, this was a single-arm study to demonstrate the feasibility of S-ESD, but not its efficacy and safety, for the treatment of colorectal neoplasms due to lack of a control arm. A comparative study is required to evaluate the treatment outcomes of S-ESD and conventional ESD in colorectal neoplasms in the next study. Furthermore, S-ESD will be applied to a wider range of the lesions, and the factors associated with treatment outcomes will be investigated. Second, all the lesions included in the study were smaller than 40 mm without fold convergence. It is reported that colorectal lesions larger than 40 mm and/or those with fold convergence are associated with a risk of ESD procedure-related complications^15^. Third, all procedures of S-ESD using the Endosaber in this study were performed by one expert endoscopist (M.E) with experience of over 200 colorectal ESDs. Insufficient operator experience is reportedly associated with incomplete resection and ESD-related perforations in colorectal ESD^20,21^. The applicability of the study outcomes to all endoscopists has not been determined even though they may be considered experts in conventional colorectal ESD. Further feasibility studies, including S-ESD for colorectal lesions larger than 40 mm operated by different endoscopists, are also required.

In conclusion, S-ESD for colorectal neoplasms smaller than 40 mm was observed to be successful with good treatment outcomes and low complication rates. S-ESD using the Endosaber could be an option for the local treatment of colorectal neoplasms.

## Methods

### Patients and study design

This prospective cohort study was conducted at the Nihon University Itabashi Hospital. The study was approved by the institution’s ethics committee (Nihon University Itabashi Hospital, Clinical Research Judging Committee, ID: RK-180911-07) and was registered in the University Hospital Medical Network Clinical Trials Registry (UMIN 000034765)^6,22^. All treatment procedures were conducted in accordance with the relevant guidelines. Written informed consent for study participation was obtained from all patients.

Consecutive patients referred to our hospital for the treatment of colorectal neoplasms were assessed for their eligibility for this study. The inclusion criteria were as follows: (1) adenomas or adenocarcinomas observed on endoscopic images or biopsies; (2) colorectal neoplasms 20–40 mm in size observed on endoscopic images, (3) patients aged between 20 and 85 years old; (4) Performance Status (Eastern Cooperative Oncology Group) 0–1; and (5) patients who could cooperate with the study. In contrast, the exclusion criteria were as follows: (1) suspected lymph node metastasis or distant metastasis based on computed tomography (CT) scans; (2) lesions with fold convergence observed on endoscopic images; (3) patients with a history of surgery for colorectal neoplasms; (4) patients with severe comorbidities; (5) patients on dialysis; (6) lesions treated by a trainee; and (7) patients who refused to participate in the study.

### S-ESD procedure

**Figure 4:**
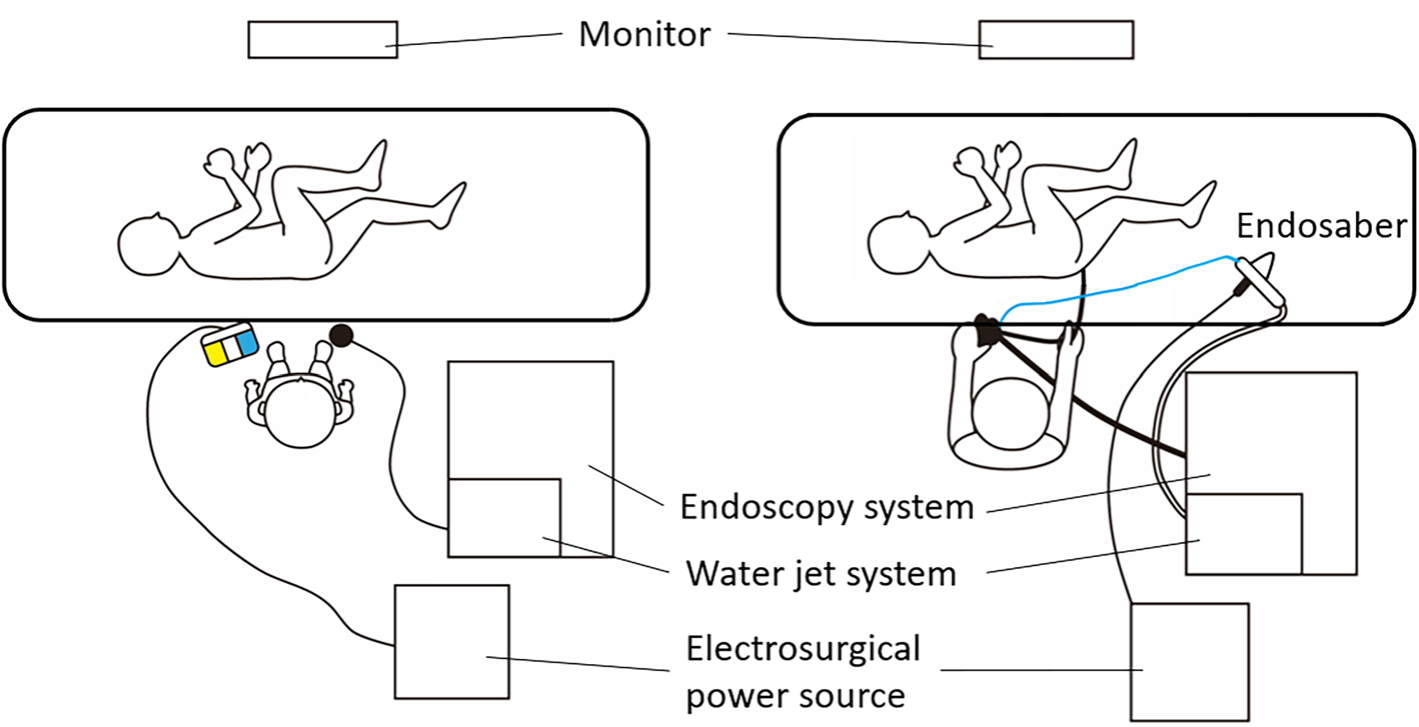
The placement of the related instruments during self-completion endoscopic submucosal dissection. The foot switch of electrosurgical power source is located at the left side of the operator. The foot switch of water jet system is located at the right side of the operator.

S-ESD was performed by a single endoscopist (M.E) with experience in performing over 200 conventional colorectal ESDs (Video 1). The endoscopist had trained in S-ESD prior to this study by performing S-ESD five times to remove mock lesions in ex vivo porcine models. The settings for the endoscopic instruments used during the S-ESD are shown in Fig. 4.

S-ESD was performed with conscious sedation using midazolam with pethidine in an endoscopic room. A lower gastrointestinal endoscope (PCF-Q260AZI, Olympus, Tokyo, Japan) attached with a disposable short-type ST hood (Fujifilm, Tokyo, Japan) was inserted under carbon dioxide insufflation. An electrosurgical current generator (VIO300D; ERBE, Tubingen, Germany) was used as a power source with its foot switch manipulated by the left foot of the operator. Glyceol (Fructose-added Glycerol; 10% glycerol, 5% fructose, and 0.9% sodium chloride) used was as an injective solution and stored in a water jet system (OFP-2; Olympus). The water jet system was connected to the Endosaber, and its foot switch was manipulated by the right foot. Injection into the submucosal layer and cleansing of the lesion in the case of bleeding are possible using this water jet system. In addition, the connection of the water jet system could be changed from the Endosaber to the endoscope as required.

**Figure 5:**
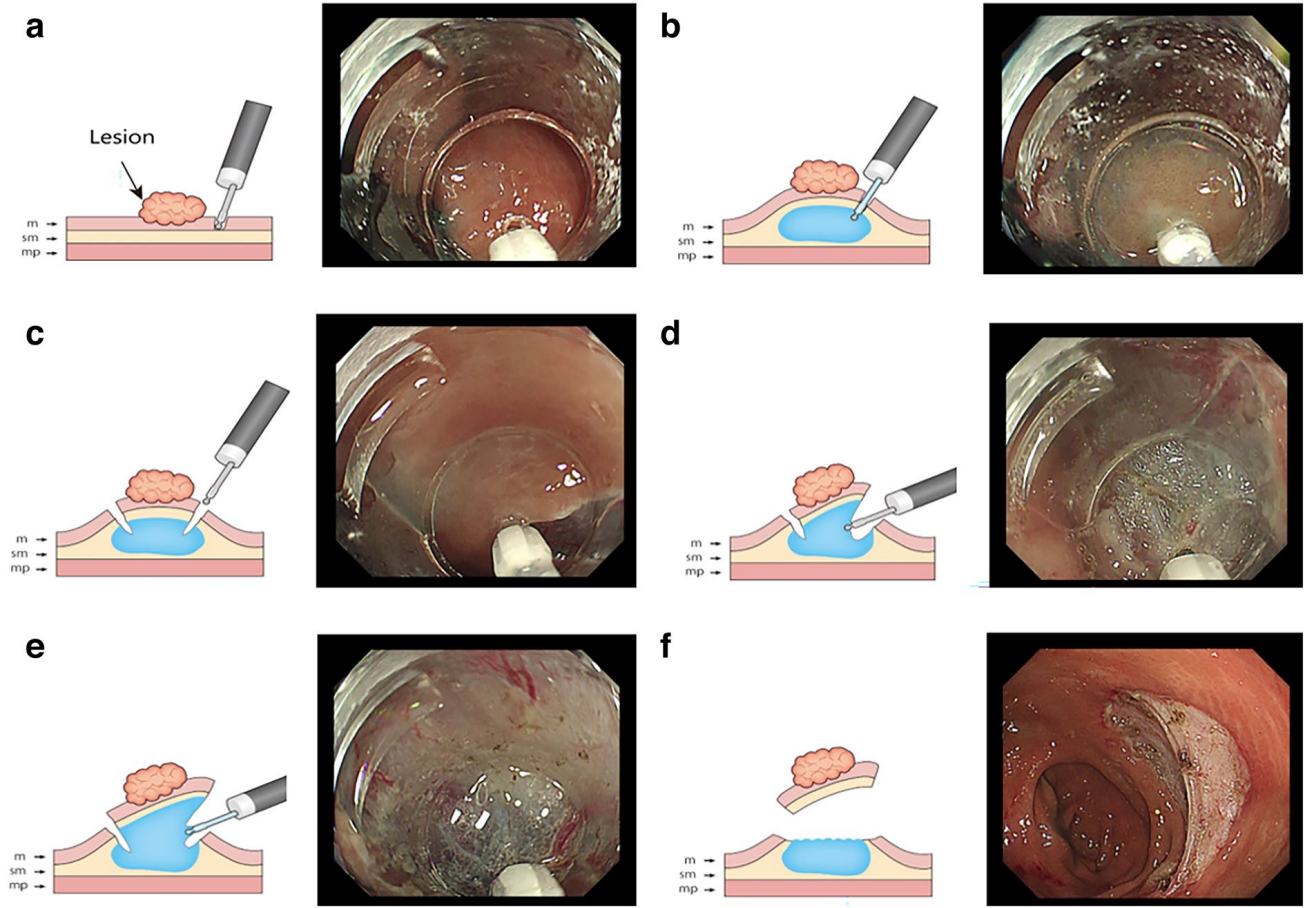
Diagrams and images depicting each stem of self-completion endoscopic submucosal dissection. m; mucosa, sm; submucosa, mp; muscularis propia. (**a**) Diagram and image depicting mucosal pre-cut. (**b**) Diagram and image illustrating submucosal injection. (**c**) Diagram and image of making an incision in the mucosal layer. (**d**) Diagram and image depicting the dissection of submucosal layer. (**e**) Diagram and image depicting additional injections into the submucosal layer. (**f**) Diagram and image depicting the removal of the lesion.

After endoscopic examination of the lesion, S-ESD using the Endosaber was performed as follows: initially, a pre-cut of the mucosa was made at the anal side of the lesion with the Endosaber in End Cut I Mode (effect 2, duration 2, interval 2) to make a pinhole for the submucosal injection (Fig. 5a). Second, Glyceol mixed with a small amount of indigocarmine dye was injected into the submucosal layer through the mucosal pinhole using the water jet function connected to the Endosaber (Fig. 5b). Third, after confirming the elevation of the lesion, a mucosal incision was initiated with the Endosaber in End Cut I Mode (effect 2, duration 2, interval 2; Fig. 5c). A mucosal flap was made at the anal side of the lesion to access the submucosal layer. Subsequently, a circumferential mucosal incision was created at approximately 5 mm outside of the lesion. Finally, the submucosal layer was dissected with the Endosaber in either Forced Coagulation Mode (effect 3, 30 W) or Swift Coagulation Mode (effect 2, 40 W; Fig. 5d). During the dissection procedure, submucosal injections were used to maintain the elevation of the lesion as needed (Fig. 5e). Submucosal dissection was continued until the lesion had been removed (Fig. 5f). Hemostasis with the Endosaber was performed if bleeding occurred during the S-ESD. During hemostasis, the Endosaber was in Forced Coagulation Mode (effect 3, 30 W). If complete hemostasis could not be achieved with the Endosaber, the use of hemostatic forceps (Coagrasper®; Olympus: Soft Coagulation Mode: effect 6, 80 W) was considered.

One medical staff member monitored the patient’s condition and sedation level and was kept on standby; assistance was required for the completion of the ESD procedures during the S-ESD. The staff member could provide extensive assistance in emergent procedures in case of complications, as required. The staff member could also assist with the procedures by adjusting the endoscopic device, electrosurgical current generator, and patient’s position.

### Histological evaluation

Resected specimens were “pinned out” onto polystyrene receivers, which were subsequently fixed in a 10% buffered formalin solution and serially sectioned as 2 mm slices. The slices were embedded in paraffin and stained with hematoxylin and eosin. Histological findings were microscopically examined by expert pathologists according to the Japanese classification of colorectal carcinoma^23^.

### Outcomes and definitions

The primary measured outcome was the success rate of the S-ESD. A successful S-ESD was defined as the completion of the S-ESD conducted by one operator using only the Endosaber. If any other devices were used, including another endo-knife, injection needle, hemostatic forceps, or hemoclips, or if any medical staff other than the operator assisted in using the device, the S-ESD was considered a failure.

The secondary outcomes included procedure time, the rates of en bloc, complete and curative resection, and the complication rate. Complication rates included the incidence of intraprocedural or delayed perforation and delayed bleeding. The procedure time was defined as the time between making the mucosal pre-cut and completion of the submucosal dissection. En bloc resection was defined as the resection of the targeted lesions in a single specimen. Complete resection was defined as resection with the horizontal and vertical margins free from colorectal neoplasms. Curative resection was defined as complete resection with the following histological conditions: (1) papillary or tubular adenocarcinomas, (2) a mucosal lesion or submucosal invasion depth of < 1000 µm, (3) no lymphovascular invasion, and (4) a tumor-budding grade of 1 (low grade)^6,24^. Intraoperative perforation was endoscopically diagnosed by observing the extramural organs or fat protruding through the colorectal wall during the S-ESD. Delayed perforation was defined as abdominal pain with free air observed in post-S-ESD X-ray or abdominal CT images. Delayed bleeding was diagnosed when patients exhibited a decrease in hemoglobin levels by > 2 g/dl or when marked hemorrhages were confirmed after the S-ESD.

### Statistical analysis

Categorical variables were expressed as numbers (%), whereas continuous variables with an abnormal distribution were expressed as medians (interquartile range [IQR]). All data analyses were performed using JMP Pro version 13.0 (SAS Institute, Cary, NC, USA).

## Supplementary Information

Supplementary Video 1.

Supplementary Legends.

## Data Availability

The datasets generated during and analyzed during the current study are available from the corresponding author on reasonable request.
