# Supplementary material for: Self-completion method of endoscopic submucosal dissection using the Endosaber for treating colorectal neoplasms (with video)
Source: Sci Rep. 2022 Apr 6;12:5821. doi: 10.1038/s41598-022-09792-8 (PMC8986775; doi:10.1038/s41598-022-09792-8)
Supplement: Supplementary file 2 — Supplementary Legends. [file 41598_2022_9792_MOESM2_ESM.docx]

**Video title**

Self-completion method of endoscopic submucosal dissection using the Endosaber for treating colorectal neoplasms

**Video legends**

Self-completion ESD using the Endosaber for treating the lateral spreading tumor of 35 mm in diameter at the rectum

ESD: endoscopic submucosal dissection
